# Delayed Cerebrospinal Fluid Rhinorrhea Associated With Ethmoidal Encephalocele After Resection of Remote Meningioma

**DOI:** 10.7759/cureus.10457

**Published:** 2020-09-14

**Authors:** Ariel M Weingarten, David M Weingarten

**Affiliations:** 1 Neuroscience, The Weingarten Institute for Neuroscience, Tamuning, GUM; 2 Neurosurgery, The Weingarten Institute for Neuroscience, Tamuning, GUM

**Keywords:** encephalocele, csf rhinorrhea, meningioma, csf fluid dynamics

## Abstract

Diagnosis and treatment of neurosurgical pathology present unique challenges in underserved areas, and many conditions may go undiagnosed, misdiagnosed, or untreated for prolonged periods. The development of an unusual complication, seemingly unrelated to an area of neurosurgical intervention, may be particularly perplexing to non-neurosurgical providers, particularly in areas where neurosurgical procedures have not historically been available. A 44-year-old male presented with a giant meningioma which was successfully resected. A nasal encephalocele was noted preoperatively but was not addressed due to lack of associated symptoms and distance from the tumor. The patient lived on a remote island and was lost to follow-up. He developed delayed cerebral spinal fluid (CSF) rhinorrhea three months after surgery, which was diagnosed and treated by local providers as allergic rhinitis for 11 months until he presented with new-onset seizure. Imaging demonstrated descent of the lateral ventricle into the encephalocele. The encephalocele was amputated and the skull base defect was repaired successfully. The alteration of ventricular anatomy and CSF fluid dynamics following tumor resection appears to have created an environment where a non-traumatic CSF leak could develop where it had previously shown no signs of developing. It may be prudent to treat skull base defects prophylactically to prevent this type of complication, particularly in patients of remote regions where regular follow-up is difficult.

## Introduction

Meningiomas are benign intracranial lesions commonly encountered by neurosurgeons, comprising approximately 25%-37% of all primary intracranial neoplasms diagnosed in the United States [1-3]. Worldwide, however, the rate of new diagnoses varies significantly based on gender, age, ethnicity, and geographical location. The rate of new cases appears to be increasing gradually, which may be attributed to the increased availability of medical care, improvements in diagnostic imaging technology, and an alteration in treatment approach for older patients [4,5].

Giant meningiomas (>5cm in diameter) are less common but may be more likely to be encountered in medically underserved areas due to lack of available resources for diagnosis or treatment, and a general trend of cultural mistrust of medical providers. Studies of racial, ethnic, and socioeconomic disparities within the United States found that minority populations and those in rural areas presented more often with advanced stages of tumor progression [6,7] or a later stage of disease in cancer cases [8-10].

Nasal encephaloceles are estimated to occur in between 1 in 5,000 and 1 in 40,000 live births [11,12], the rate being heavily influenced by the region of the world with Southeast Asia having the highest prevalence. The genetics and epidemiology of the Pacific Islander populations have not been as thoroughly explored, but many Pacific Islanders have Southeast Asian ancestry. Some defects are not outwardly visible and may be asymptomatic, leading to prolonged delays in diagnosis and treatment. Patients presenting with adult-onset seizures are sometimes found to have undiagnosed encephaloceles [12-15].

We report here a patient who had an asymptomatic ethmoidal encephalocele prior to resection of a giant meningioma, who then developed a delayed cerebral spinal fluid (CSF) leak from the encephalocele months after tumor resection.

## Case presentation

A 44-year-old male presented in the emergency department with a three-year history of progressively worsening ataxia, apathy, and aphasia. Computed tomography (CT) scan had revealed a large left frontal lesion a year prior, but the patient had declined surgical intervention at that time. A CT scan performed three years prior had shown no evidence of lesion, demonstrating explosive growth within a three-year period. Records from the earliest scan are not available and the patient does not recall the indication for the scan. On examination, he had profound expressive aphasia. For receptive language, he required some translation by family members, who reported that he had previously been fluent in English, but was now limited to Chamorro, his first language. He had right-sided hyperreflexia and ataxia.

**Figure 1:**
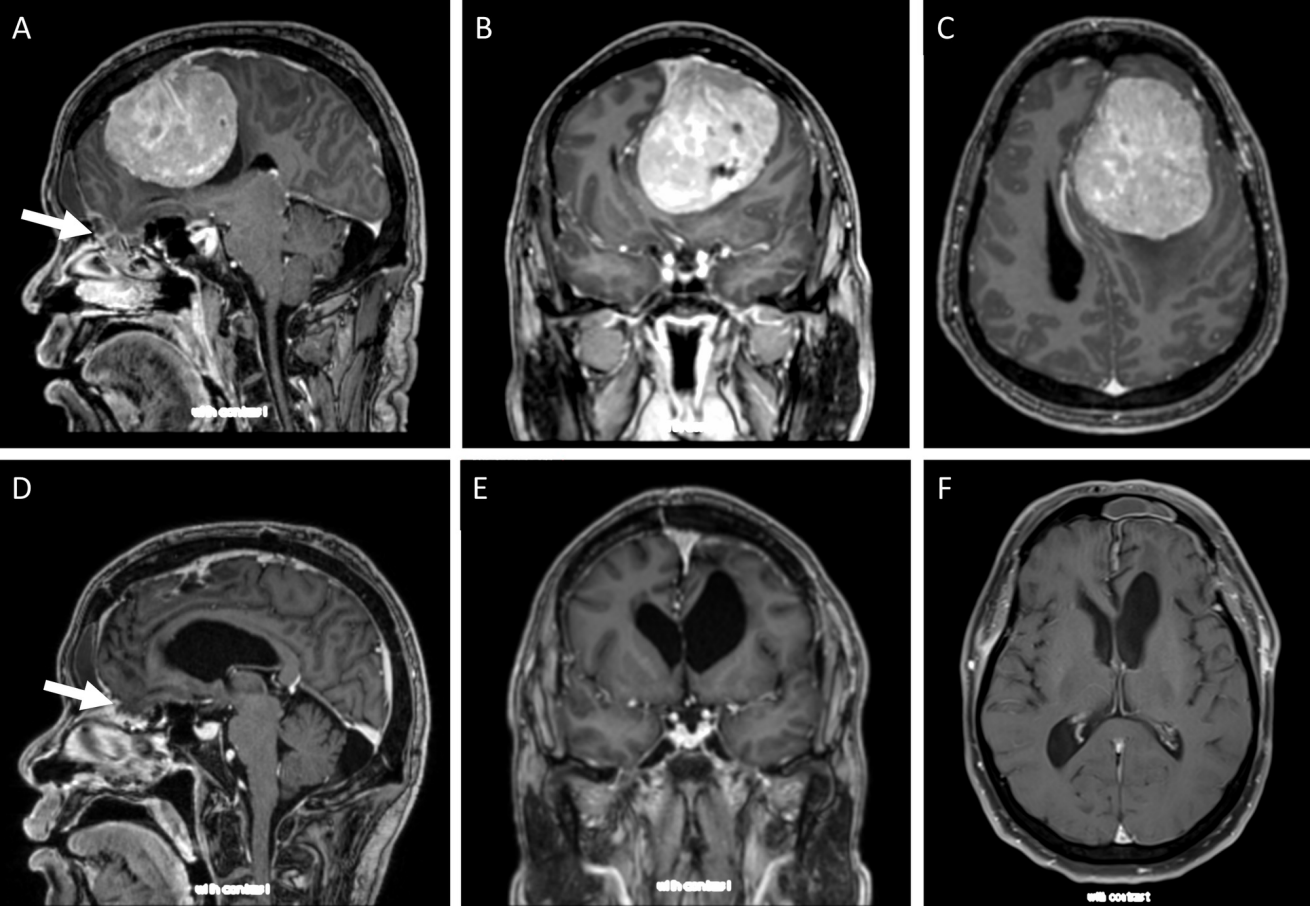
(A-C) Preoperative and (D-F) postoperative MRI of giant meningioma. Note small encephalocele (white arrows), remote from resection bed. MRI, magnetic resonance imaging

Magnetic resonance imaging (MRI) showed a 7.7 x 6.3 x 6.2cm lesion consistent with a giant meningioma (Figure 1). A nasal encephalocele was also noted, remote from the tumor. The island lacks neuro-endoscopy equipment, and hence endoscopic evaluation and treatment of the encephalocele and skull base defect were not feasible. The patient had no evidence of CSF leak and had neither signs nor history of meningitis; the encephalocele was thus felt to be chronic and asymptomatic, and was not addressed at the time of tumor resection.

Left frontal craniotomy was performed, and a gross total resection was achieved. The patient’s preoperative ataxia resolved quickly, and he was ambulatory without assistive devices by the time staples were removed. His aphasia similarly improved. No formal cognitive testing was performed, but he appeared to still have some residual cognitive impairment. The patient's family confirmed that he had returned to his cognitive baseline. He had two months of follow up, but then returned to his home island of Saipan where he was lost to follow up despite multiple attempts to reach him. Tumor pathology was determined to be WHO grade I meningioma.

**Figure 2:**
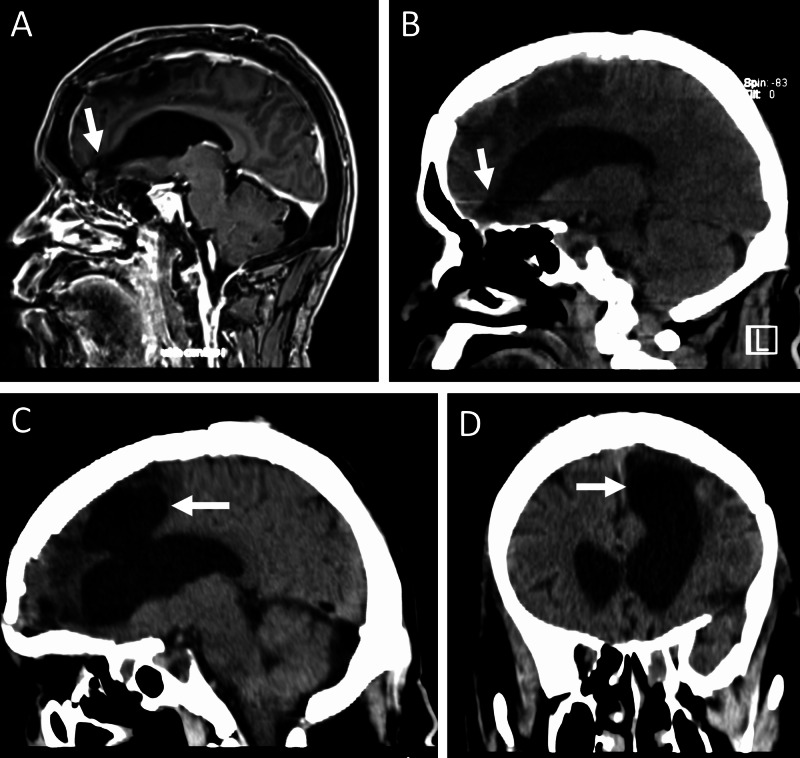
(A) MRI and (B) CT showing ventricle descending into defect in cribriform plate; (C) sagittal and (D) coronal CT showing ballooning of ventricle into previous resection cavity (not seen on scans post-tumor resection). CT, computed tomography; MRI, magnetic resonance imaging

**Figure 3:**
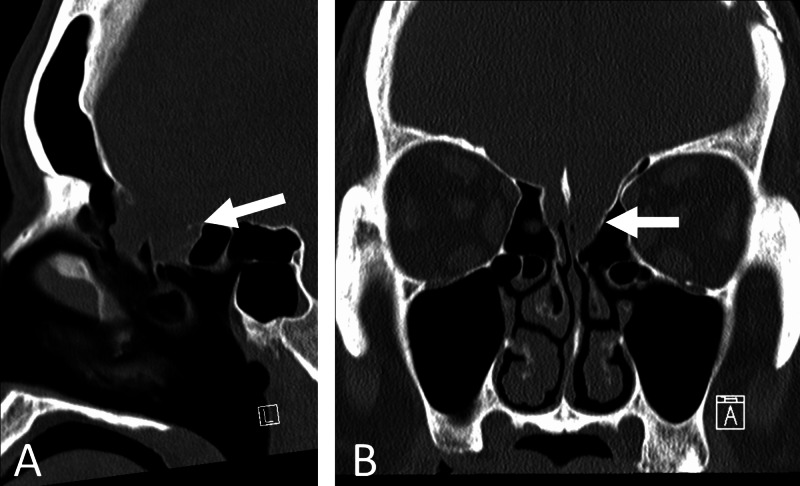
(A) Sagittal and (B) coronal bone windows of CT demonstrating bony defect in cribriform plate. CT, computed tomography

Fourteen months postoperatively, he was readmitted to the emergency room in Saipan with seizures and persistent rhinorrhea; he had developed the rhinorrhea approximately three months after surgery and had been prescribed a nasal steroid spray for allergic rhinitis. Clinical testing revealed a robust flow of clear fluid from his left nostril. CT and subsequent MRI scans demonstrated descent of the left lateral ventricle into the known osseous defect in the cribriform plate (Figures 2, 3). Ventricular anatomy had been normal on his CT three years prior and had later become compressed by the tumor. Notably, there had been no sign of ventricular herniation into the encephalocele prior to the meningioma or during its growth. At no point prior or subsequent to his tumor resection did he display any of the typical clinical signs of hydrocephalus.

Given the lack of endoscopic equipment, the skull base defect was repaired via open craniotomy. The multi-pedunculated encephalocele was found to be emerging through 2-3mm dural defects. These were suspected to be nonfunctional based on the miniscule pedicles from which they emerged and were hence amputated. The defect was packed with fat graft, and a titanium mesh was secured over the ethmoidal defect. The frontal sinus was cranialized, stripped of mucosa and packed with fat. A vascularized pericranial flap was secured over the mesh. A lumbar drain was placed and, given the risk of meningitis from such a prolonged CSF leak, CSF was sampled for laboratory testing and culture. The patient was kept on broad-spectrum antibiotics until labs were available. Remarkably, no signs of infection were found and the patient showed no clinical signs of meningitis. A clamp trial was attempted, but a slight leak persisted, and hence the drain was re-opened. The patient accidentally removed his lumbar drain seven days postoperatively, but no further leak was observed and the drain was not replaced. Given his previous disappearance to follow up, he was kept on island in a hotel until three months post-operatively. At that time, he was doing well with no evidence of CSF leak and was released to return to Saipan. Pathology of the encephalocele showed no neoplasm or acute inflammation. Postoperative imaging following skull base defect repair showed that the previously descended ventricle had ballooned up into the resection cavity (Figure 2), but serial imaging did not show any progression of this dilation, and at no time before or after skull base repair did he display any clinical signs of hydrocephalus.

## Discussion

Giant meningiomas and frontoethmoidal encephaloceles, while certainly recognized throughout the world, are each relatively uncommon entities in their own right [1-3,11,12]. In underserved areas, both diagnostic and treatment delays may result in an increase in late-stage disease rates [6-10]. CSF leakage from long-silent encephaloceles is also a known phenomenon [12-15], but is usually preceded by polypectomy or biopsy of nasal mass [16]. Our patient had lived 45 years of his life without developing such a leak. His meningioma was not near the encephalocele, and there was no suggestion of ventricular descent into the encephalocele. There was thus no preoperative suggestion that such leak was a substantial risk. We consider several potential hypotheses to explain this development. It is possible that the large meningioma increased intracranial pressure such that the cribriform plate was weakened, but the tumor itself blocked flow of CSF from the ventricles into this newly created path of least resistance. Traction on the encephalocele during meningioma resection could have disrupted the integrity of the encephalocele tissue, leaving route for CSF egress. Postoperative hydrocephalus could develop after resection of a large tumor and could contribute to forcing CSF out through the skull base defects.

These explanations are of course speculative, and our case illustrates elements which might suggest an alternative hypothesis. First, the CSF leak was delayed by several months postoperatively, which speaks against an immediate injury such as traction. Second, immediately after the meningioma resection, the operative cavity collapsed. It did not balloon out, as would be expected if intracranial pressure were high. As the ventricle descended into the encephalocele, the large resection cavity did not dilate, as would be predicted if it were a purely pressure-related phenomenon. One might anticipate that the cavity would initially fill with CSF and then the encephalocele might present a point of weakness as the previously compressed brain tissue expanded and the resection cavity collapsed, but the order of events suggests a more complex phenomenon: the resection cavity immediately collapsed; the ventricle slowly descended into the encephalocele without dilating the resection cavity; the resection cavity then ballooned out when the encephalocele was repaired, without any other clinical or radiographic signs of hydrocephalus.

CSF leaks not related to a traumatic event are rare occurrences, but have been related to the presence of neoplasms, especially skull-base tumors, inflammation, basal encephaloceles, benign intracranial hypertension or pseudotumor cerebri [17,18]. In this case, we do not see evidence pointing to any of these as a direct cause. The CSF fluid dynamics model proposed by Levine and Nejat [19] may help to explain the unusual development of this delayed CSF leak. Due to a preexisting skull base defect and subsequent alteration in ventricular anatomy, a new pattern of CSF flow may provide sufficient hydrodynamic forces to herniate the now-uncompressed ventricle into the encephalocele. We thus hypothesize that alteration in brain and ventricular anatomy due to massive tumor growth and resection thereof caused changes in CSF flow dynamics such that, over a three-month period, the ventricle descended into the encephalocele and ruptured through it, causing CSF leakage.

## Conclusions

This case highlights some of the difficulties in treating neurosurgical patients in remote regions of the world. The “standards of care” can vary depending on the availability of trained medical personnel and adequate facilities. This patient had a substantially delayed complication which went unrecognized due to the absence of neurosurgical resources in his area. For patients with asymptomatic skull base defects, it is important to take into account the potential alteration of CSF fluid dynamics when performing other, seemingly unrelated procedures. Compliance with follow-up may also be problematic when it requires travel to a remote care facility, and may prevent timely diagnosis of complications, as in this case. The presence of skull base defects should prompt aggressive counseling about the importance of follow-up. In extreme cases where patients may be unable to schedule a regular follow-up, it may be appropriate to treat such lesions prophylactically, even when they seem asymptomatic and unrelated to the primary lesion at hand.

## References

[1] Claus EB, Bondy ML, Schildkraut JM, Wiemels JL, Wrensch M, Black PM (2005). Epidemiology of intracranial meningioma. Neurosurgery.

[2] Ostrom QT, Gittleman H, Xu J, Kromer C, Wolinsky Y, Kruchko C, Barnholtz-Sloan JS (2016). CBTRUS statistical report: primary brain and other central nervous system tumors diagnosed in the United States in 2009-2013. Neuro-Oncology.

[3] Ostrom QT, Gittleman H, Truitt G, Boscia A, Kruchko C, Barnholtz-Sloan JS (2018). CBTRUS statistical report: primary brain and other central nervous system tumors diagnosed in the United States in 2011-2015. Neuro-Oncology.

[4] Fisher JL, Schwartzbaum JA, Wrensch M, Wiemels JL (2007). Epidemiology of brain tumors. Neurol Clin.

[5] Bondy ML, Scheurer ME, Malmer B (2008). Brain tumor epidemiology: consensus from the Brain Tumor Epidemiology Consortium. Cancer.

[6] Curry WT, Carter BS, Barker FG (2010). Racial, ethnic, and socioeconomic disparities in patient outcomes after craniotomy for tumor in adult patients in the United States, 1988-2004. Neurosurgery.

[7] Curry WT, Barker FG (2009). Racial, ethnic and socioeconomic disparities in the treatment of brain tumors. J Neuro-Oncol.

[8] Silverstein MD, Nietert PJ, Ye X, Lackland DT (2002). Access to care and stage at diagnosis for patients with lung cancer and esophageal cancer: analysis of the Savannah River Region Information System cancer registry data. South Med J.

[9] Smith-Gagen J, White LL, Santos A, Hasty SM, Tung WC, Lu M (2019). Scope-of-practice laws and expanded health services: the case of underserved women and advanced cervical cancer diagnoses. J Epidemiol Commun Health.

[10] Kim SJ, Glassgow AE, Watson KS, Molina Y, Calhoun EA (2018). Gendered and racialized social expectations, barriers, and delayed breast cancer diagnosis. Cancer.

[11] Tirumandas M, Sharma A, Gbenimacho I, Shoja MM, Tubbs RS, Oakes WJ, Loukas M (2013). Nasal encephaloceles: a review of etiology, pathophysiology, clinical presentations, diagnosis, treatment, and complications. Child's Nervous Syst.

[12] Wilkins RH, Radtke RA, Burger PC (1993). Spontaneous temporal encephalocele: case report. J Neurosurg.

[13] Morone PJ, Sweeney AD, Carlson ML (2015). Temporal lobe encephaloceles: a potentially curable cause of seizures. Otol Neurotol.

[14] Hyson M, Andermann F, Olivier A, Melanson D (1984). Occult encephaloceles and temporal lobe epilepsy: developmental and acquired lesions in the middle fossa. Neurology.

[15] Whiting DM, Awad IA, Miles J, Chou SS, Lüders H (1990). Intractable complex partial seizures associated with occult temporal lobe encephalocele and meningoangiomatosis: a case report. Surg Neurol.

[16] Dempsey PK, Harbaugh RE (1988). Encephalomeningocele presenting with spontaneous cerebrospinal fluid rhinorrhea in an elderly man. Neurosurgery.

[17] Schuknecht B, Simmen D, Briner HR, Holzmann D (2008). Nontraumatic skull base defects with spontaneous CSF rhinorrhea and arachnoid herniation: imaging findings and correlation with endoscopic sinus surgery in 27 patients. Am J Neuroradiol.

[18] Bhattacharjee S, Reddy DC, Chatterjee S, Choudhury B (2018). Anteromedial temporal encephalocele: a rare cause for spontaneous cerebrospinal fluid rhinorrhoea. J Marine Med Soc.

[19] Levine D, Nejat R (2014). Hydrodynamic herniation: pathophysiology of brain, spinal cord and nerve displacements associated with leakage or diversion of cerebrospinal fluid. Curr Trends Neurol.

